# Economic evaluation of an experience sampling method intervention in depression compared with treatment as usual using data from a randomized controlled trial

**DOI:** 10.1186/s12888-017-1577-7

**Published:** 2017-12-29

**Authors:** Claudia J. P. Simons, Marjan Drukker, Silvia Evers, Ghislaine A. P. G. van Mastrigt, Petra Höhn, Ingrid Kramer, Frenk Peeters, Philippe Delespaul, Claudia Menne-Lothmann, Jessica A. Hartmann, Jim van Os, Marieke Wichers

**Affiliations:** 1 Department of Psychiatry and Psychology, School of Mental Health and Neuroscience, Maastricht University Medical Centre, Maastricht, The Netherlands; 2 GGzE, Institute for Mental Health Care Eindhoven and De Kempen, Eindhoven, The Netherlands; 3 Department of Health Services Research, School of Public Health and Primary Care (CAPHRI), Maastricht University, Maastricht, The Netherlands; 4 Trimbos Institute, Netherlands Institute of Mental Health and Addiction Department of Public Mental Health, Utrecht, The Netherlands; 5 Mondriaan Mental Health Trust South Limburg, Heerlen, The Netherlands; 6 Orygen, the National Centre of Excellence in Youth Mental Health, University of Melbourne, Melbourne, Australia; 7 Department Psychiatry, Brain Centre Rudolf Magnus, Utrecht University Medical Centre, Utrecht, the Netherlands; 8 King’s College London, King’s Health Partners Department of Psychosis Studies; Institute of Psychiatry, London, UK; 9 Interdisciplinary Center Psychopathology and Emotion regulation (ICPE), Department of Psychiatry, University Medical Centre Groningen (UMCG), Groningen, The Netherlands

**Keywords:** Cost-effectiveness analysis, Cost-utility analysis, Ecological momentary assessment, Experience sampling method, Intervention study, Psychological feedback, Depressive disorder

## Abstract

**Background:**

Experience sampling, a method for real-time self-monitoring of affective experiences, holds opportunities for person-tailored treatment. By focussing on dynamic patterns of positive affect, experience sampling method interventions (ESM-I) accommodate strategies to enhance personalized treatment of depression―at potentially low-costs. This study aimed to investigate the cost-effectiveness of an experience sampling method intervention in patients with depression, from a societal perspective.

**Methods:**

Participants were recruited between January 2010 and February 2012 from out-patient mental health care facilities in or near the Dutch cities of Eindhoven and Maastricht, and through local advertisements. Out-patients diagnosed with major depression (*n* = 101) receiving pharmacotherapy were randomized into: (i) ESM-I consisting of six weeks of ESM combined with weekly feedback regarding the individual’s positive affective experiences, (ii) six weeks of ESM without feedback, or (iii) treatment as usual only. Alongside this randomised controlled trial, an economic evaluation was conducted consisting of a cost-effectiveness and a cost-utility analysis, using Hamilton Depression Rating Scale (HDRS) and quality adjusted life years (QALYs) as outcome, with willingness-to-pay threshold for a QALY set at €50,000 (based on Dutch guidelines for moderate severe to severe illnesses).

**Results:**

The economic evaluation showed that ESM-I is an optimal strategy only when willingness to pay is around €3000 per unit HDRS and around €40,500 per QALY. ESM-I was the least favourable treatment when willingness to pay was lower than €30,000 per QALY. However, at the €50,000 willingness-to-pay threshold, ESM-I was, with a 46% probability, the most favourable treatment (base-case analysis). Sensitivity analyses confirmed the robustness of these results.

**Conclusions:**

We may tentatively conclude that ESM-I is a cost-effective add-on intervention to pharmacotherapy in outpatients with major depression.

**Trial registration:**

Netherlands Trial register, NTR1974.

**Electronic supplementary material:**

The online version of this article (10.1186/s12888-017-1577-7) contains supplementary material, which is available to authorized users.

## Background

Depression consistently ranks high worldwide in terms of disability [1–3] and societal costs due to health care consumption and productivity loss [4]. In the Netherlands, twelve-month prevalence of a depressive disorder is 5.2% [5], health care costs are estimated at 1592 million euros (1.8% of the total health care costs in 2011) [6], and disability days are eight times higher compared with the general population [7].

Because of the high disease burden of depression [1–5], non-pharmacological interventions that can enhance (psychopharmacological) treatment effects have the potential to be cost-effective. Although clear evidence exists for the effectiveness of combined pharmacotherapy with psychotherapy in the treatment of depression [8], face-to-face psychological treatment is cost-intensive and may, unfortunately, not be routinely available. Furthermore, it is estimated that optimal use of cognitive-behavioural therapy, counselling, and medication would lower the disease burden of depression by 35% at most [9]. Thus, efforts to improve the efficacy of pharmacotherapy combined with psychotherapy are considered a priority.

Moment-to-moment ambulatory monitoring tools ―designed to collect real-life data with easy and immediate availability to both patients and professional caregivers― pave the road for potential low-cost strategies to improve and *personalize* mental health care. In particular, digitalized experience sampling method (ESM) tools incorporating repeated in-the-moment assessments of affective experience and context seem to be an acceptable and feasible strategy to provide unique person-tailored insights about affective patterns in daily life [10–12]. Interventions using the Experience Sampling Method (Experience Sampling Method-Interventions or ESM-I) may, therefore, provide possibilities for mobile health (mHealth) interventions in depression [13]. These interventions could be directed at increasing positive affect, as ESM studies have shown that a high ability to experience positive affect may predict development, course, and recovery of depression [14–16].

A first effect study showed that ESM-I as add-on intervention to psychopharmacological treatment, with feedback focussed on positive affect, was efficacious in reducing symptoms in patients with depression [17]. Although some evidence exists that ambulatory self-assessments may be cost-effective tools to manage health conditions [18, 19], to our knowledge, no randomized controlled trials have investigated the cost-effectiveness or cost-utility of any ESM-interventions in patients with depression. The present paper presents a trial-based economic evaluation using data from a randomised controlled trial. The purpose is to evaluate the cost-effectiveness and cost-utility of ESM-I as add-on intervention to psychopharmacological treatment as usual, from a societal perspective. Because the hypothesis was that ESM-derived feedback on daily life patterns is an essential ingredient, ESM-I was compared with two control conditions: (1) ESM self-monitoring without feedback, hereafter pseudo-intervention; and (2) treatment as usual (hereafter control group).

## Method

### Setting

For the current randomized controlled trial [17], participants were recruited between January 2010 and February 2012 from out-patient mental health care facilities in or near the Dutch cities of Eindhoven and Maastricht, and through local advertisements.

Patients were considered eligible if they were aged between 18 and 65 years; diagnosed with major depression according to DSM-IV [20] with current or residual symptoms (score of >7 on the 17-item Hamilton Depression Rating Scale (HDRS) [21]); and treated with antidepressants or mood stabilizers. Patients were excluded if they met criteria for a non-affective psychotic disorder according to DSM-IV or if they met criteria for a manic, hypo-manic or mixed episode within the past month.

The study was approved by an institutional review board (Medical Ethics Committee of Maastricht University Medical Centre); all participants provided written informed consent before enrolment. The trial was registered in the Netherlands Trial Register (ID: NTR1974). The study was performed according to the declaration of Helsinki. The original protocol and a CONSORT checklist are provided (see Additional files 1 and 2).

### Treatment arms

A randomized controlled trial was conducted with three treatment arms [17, 22]. All participants were asked to complete a five-day ESM baseline assessment. After baseline, patients were randomly allocated to the ESM-I, pseudo-intervention, or control group. Randomization (allocation ratio 1:1:1) was stratified for duration of pharmacological treatment (use of a particular antidepressant for shorter vs. longer than 8 weeks prior to study entry) and psychotherapy (yes/no). After all baseline assessments were performed, allocation took place using opaque, sealed, sequentially numbered envelopes (prepared by an independent research coordinator) with a number sequence produced by an electronic random sequence generator (http://www.random.org), in blocks of six. Envelopes were opened by the researcher (CS, PH, IK, CML, JH) or a research assistant. Allocation was not blinded.

The ESM-I group participated in an ESM procedure (three days per week over a six-week period; see below), as addition to treatment as usual. This group received weekly standardised *feedback on personalized patterns of positive affect.* The pseudo-intervention group participated in the same ESM procedure but received *no feedback*. The control group received no additional intervention (treatment as usual).

### Experience sampling method

ESM was carried out in accordance with previous studies [11, 23–25]: participants received a dedicated electronic ESM device (‘PsyMate’, [26]) which emitted a signal at a random moment in each of ten 90-min time blocks between 07:30 am–10:30 pm, prompting participants to fill in self-assessments including current positive and negative affect, activities, and context (7-point Likert scale ratings and forced-choice questions).

### Intervention

For 6 consecutive weeks, ESM-I participants engaged in ESM self-monitoring for three consecutive days within each week. Each ESM week was followed by a face-to-face feedback session with one of the researchers (a psychologist or psychiatrist, *n* = 5). These six sessions were held at the participating mental health institutions or at Maastricht University. In these sessions, the researcher provided the participant verbal, graphical, and written feedback using the participant’s ESM data, delivered according to a fixed format, in a fixed order. Feedback showed actual levels of positive affect in the context of daily life activities, events, and social situations. In addition, changes in positive affect level and depressive feelings over the course of the ESM intervention were visualized (see [17] for examples). A bullet-point summary report of the feedback was also given to both the participant and his/her mental health professional using a fixed template.

The procedure in the pseudo-intervention group was identical to the procedure in the ESM-I group except that no feedback was given. In the pseudo-intervention group, sessions were filled with an alternative activity (an HDRS interview) to keep duration of contacts equivalent to the ESM-I group.

### Treatment as usual

Treatment as usual consisted of psychopharmacological treatment as usual, either in primary or ambulatory specialized care, that is, patients were treated with antidepressants or mood stabilizers, either as stand-alone treatment (e.g., with supportive counselling) or in combination with psychotherapeutic treatment.

### Procedure

Full screening occurred two weeks before randomization to treatment. Severity of symptoms was assessed to determine eligibility. In addition, self-report instruments were completed assessing costs and quality adjusted life years (QALYs). One week later, the ESM procedure was explained and all participants engaged in a five-day ESM procedure after which baseline assessment took place. At baseline, symptoms were assessed and participants were subsequently randomized to treatment. Assessment of costs and QALYs was not repeated at baseline, thus full screening was used as a proxy for baseline. The 6-week intervention period (week 1–6) was followed by an immediate post-assessment, including symptom assessments and a five-day ESM post-assessment (week 7), and a first follow-up (week 8). Other follow-up assessments were conducted at 4 (week 12), 8 (week 16), 12 weeks (week 20), and 24 weeks (week 32) after this first follow-up assessment. At follow-up, symptoms (all follow-up assessments), costs, and QALYs (follow-up at week 20 and 32) were assessed.

### Outcome measures

In the cost-effectiveness analyses, the Hamilton Depression Rating Scale-17 (HDRS) [21] was used as the primary outcome measure. The HDRS is a semi-structured interview measuring the severity of depressive symptoms over the past week. A higher HDRS score indicated higher levels of depression. In the bootstrapping models (see Statistical analysis paragraph), the HDRS was reversed (higher score is better outcome, as is obligatory in economic evaluations). Symptomatic remission was obtained using the HDRS; participants with a HDRS score ≤ 7 were considered to be in symptomatic remission [27].

In the cost-utility analyses, QALYs were used as the primary outcome. QALYs were generated for each participant, based on health states. These health states were obtained using the EuroQol-5D-3 L (EQ-5D; [28]), a generic, self-report instrument. At the start of the trial, this was the most recent version of the EQ-5D. Utilities for each possible health state were available from a UK general population survey, which is the international standard to valuate the EQ-5D [29, 30]. Those utilities scores were used as weights to obtain quality adjusted life years (QALYs) [31].

### Economic evaluation

#### Study perspective and time horizon

The economic evaluation was performed from a societal perspective, including intervention costs, health care costs, as well as productivity losses. The time horizon (i.e. the period of time evaluated in the analyses [32]) for this study was 32 weeks, equalling the full assessment period (eight weeks until end of intervention plus 24 weeks follow-up). As the time horizon was <1 year, no discounting of costs and effects was necessary (future costs and benefits were not valued to the present). All costs were presented in Euro’s and calculated to their 2012 value using price index figures from Statistics Netherlands [33]. The most recent cost prices that were available in 2012 (the year the trial ended) were used to calculate costs.

#### Costs measures and valuation

In the cost-assessment, we a priori identified health care costs (See Additional file 3: Tables S1 and S2 for details), absence from work (absenteeism), and productivity loss at work (presenteeism) as relevant. Information on costs was monitored with two self-report instruments assessing health care consumption, absence from work, and productivity loss at work in the past three months. Health care consumption and medication use were assessed using the Trimbos/Institute for Medical Technology Assessment questionnaire for Costs associated with Psychiatric Illness (TiC-P; [34]); the Productivity and Disease Questionnaire (PRODISQ; [35, 36]) was used to measure costs of absence from work and cost of productivity loss at work.

The valuation of health care costs was based on the updated Dutch Manual for Cost Analysis in Health Care Research [37]. This manual contains methods and standard cost prices for economic research in health care. The costs of medication were based on the Dutch medication prices [38].

Costs of absenteeism were calculated using the human capital method by multiplying the number of days absent with an estimation of the productivity costs per hour of each participant (obtained from age and gender specific productivity costs also including an elasticity factor of 0.8; elasticity to account for the proportional reduction in productivity resulting from absence from work) ([37], Table 6.1). Productivity loss (presenteeism) was calculated using the QQ method, that is 100% − [quantity of work] × [quality of work] [39, 40]. This percentage was multiplied by the number of working hours in 3 months (385 h [37]), because the PRODISQ was assessed every three months (or data were imputed; see Statistical analysis).

#### Intervention costs

Cost calculations of the intervention were based on a psychologist salary (to translate the researchers time delivering the intervention to the salary of a health care professional), which was €173 per hour in 2009 [37], calculated to the 2012 value of €183.73. ESM-I and pseudo-intervention group participants completed on average 5.3 (*SD* = 1.7) and 5.7 (*SD* = 0.94) intervention sessions, respectively. Mean duration of these sessions was 48.9 and 39.5 min. Thus, total time spent per patient was 4.3 h (€792.62) for ESM-I, and 3.7 h (€689.45) for the pseudo-intervention.

#### Willingness-to-pay threshold

In the Netherlands, proportional upper limits exist for various levels of severity: €20,000 (mild condition), €50,000 (moderate severe condition), €80,000 (severe condition) [41]. It is unclear which of these limits would be most appropriate to set as a maximum willingness to pay for the QALY for patients with major depression. In most previous economic evaluations, the willingness-to-pay threshold for depression was set above this threshold for mild conditions toward the threshold for moderate severe conditions (e.g., [42–44]). Therefore, we set the willingness-to-pay threshold for ESM-I in major depression at €50,000 ($59,115).

### Statistical analysis

Power calculations using the STATA SAMPSI command were based on previous work [45], and led to an initial sample size of 120 with a power of 84% to detect a 3-point difference in the score on the 17-item HDRS [46, 47], the primary effectiveness outcome [17]. However, because many participants were excluded, the inclusion rate was lower than expected. The eventual number of patients who participated in the trial was 102.

The economic evaluation consisted of a base-case cost-effectiveness and cost-utility analysis, and sensitivity analyses. For the main analysis (base-case), intention-to-treat (ITT) data were used. All analyses in step 1–4 were performed using Stata version 13 [48], the economic evaluation in step 5 was performed using Microsoft Excel (version 2010).

First, missing observations at the 32-weeks assessment were replaced by Last Observation Carried Forward; missing observations at the 20-weeks assessment were replaced by Next Observation Carried Backward. Second, costs and QALYs of week 1–8 were estimated by individual mean imputation of the baseline and the week 20 assessment (corrected for length of the period by multiplying by 0.667) to obtain information of the full 32-week period [47]. Subsequently, a total cost variable was generated, being the sum of all above-mentioned costs in the full 32 weeks (base-case societal perspective). Similarly, QALYs were summed for the full 32-week period. Third, costs and QALYs over 32-weeks and HDRS at 32 weeks were analysed using linear regression analysis to provide background information to the economic evaluation results. These regression models included treatment arm as well as baseline values of the dependent variable (costs, QALYs, and HDRS scores, respectively) as independent variables and included all assessments of all participants (intention-to-treat). When costs were the dependent variable, assumptions for linear regression were not met and *p*-values were obtained using permutation analysis. Fourth, data were prepared for inclusion in the fifth step (the base-case analyses of the economic evaluation): costs were controlled for baseline costs using the delta method [49], i.e., baseline costs were subtracted from total costs post-assessment. Coefficients obtained from a regression analysis corrected for baseline costs could not be used in the present data, because the data failed to meet the assumption of a normal distribution of the cost residuals, even after applying methods to address outliers [49] (see also Methodological considerations). HDRS scores and QALYs were exported to Excel without further transformations.

For the fifth step, cost and effect pairs per participant were imported in a previously designed Excel file. Because residuals in the analysis were not normally distributed, non-parametric bootstrap resampling techniques were used to explore sample uncertainty around estimates of the cost-utility and cost-effectiveness analysis, using the original data of the three treatment groups. Using this Excel file, 5000 replications were generated. We calculated incremental cost-effectiveness ratios (ICER) by dividing the incremental costs by the incremental effects (HDRS scores); by dividing the incremental costs by the differences in QALYs, we calculated incremental cost-utility ratios (ICUR). Using the 5000 replications, cost-effectiveness acceptability curves (CEAC) were generated [31, 50]. For QALYs, an a priori willingness-to-pay threshold of €50,000 was defined (see above).

### Sensitivity analyses

Several one-way sensitivity analyses for deterministic variables were performed to assess how sensitive results are to different input values in a priori selected parameters. First, QALYs were based on the Dutch tariff [51] rather than the UK tariff [29]. Second, costs for a standard GP-contact (€29.73 in 2012) were replaced by costs of a psychiatric GP contact (€60.53) as obtained from the cost manual [37]. Third, the economic evaluation was performed from a health care perspective, rather than a societal perspective. Fourth, complete cases were analysed (as opposed to intention-to-treat). Finally, analyses were performed when NOT adjusting for baseline costs.

## Results

### Participants and baseline characteristics

**Fig. 1:**
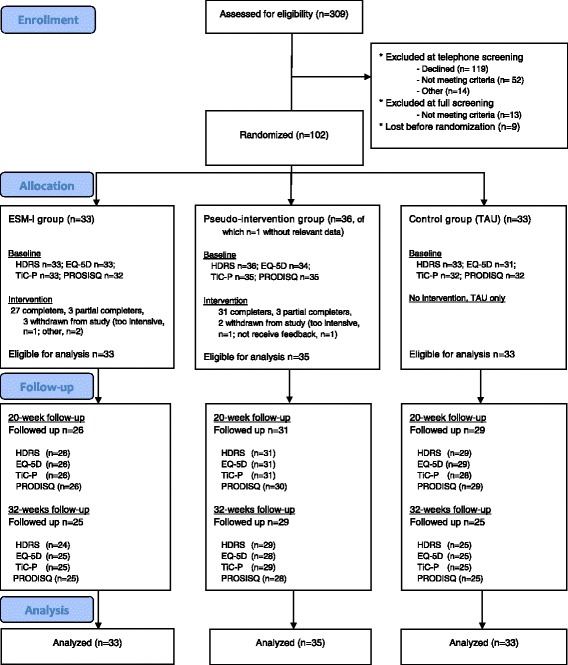
Flow diagram of the study. ESM = Experience Sampling Method; TAU = treatment as usual HDRS – 17-item Hamilton Depression Rating Scale; EQ-5D – EuroQol-5D-3 L; TiC-P – Trimbos/IMTA questionnaire for Costs associated with Psychiatric Illness; PRODISQ – The Productivity and Disease Questionnaire

A total number of 102 participants were randomized to one of the three treatment arms (see participant flow in Fig. 1). The intention-to-treat sample consisted of 101 participants (*n* = 33 ESM-I, *n* = 35 pseudo-intervention, *n* = 33 control group), given that one participant randomized to the pseudo-intervention did not fill in any of the assessments needed for the present analyses, not at baseline, nor at follow-up. At the 20-week and 32-week follow-up assessments, 86 (85%) and 80 (79%) participants responded, respectively. Table 1 presents baseline characteristics (see [17] for more details). At baseline, mean HDRS score was 2 units lower in the ESM-I group than in the control group, and total costs (societal perspective over 3 months) were about €450 lower (Table 1). Control group participants more often had a bipolar disorder, more often had a recent switch in their antidepressant medication, and less often received psychotherapy compared with the other two groups; pseudo-intervention group participants more often had a comorbid axis I disorder and were more often treated in primary health care (Table 1).

**Table 1:** Baseline characteristics

	ESM-I (*n* = 33)	Pseudo-intervention (*n* = 35)	Control group (*n* = 33)
Gender, male: n (%)	17 (51.5)	14 (40.0)	15 (45.5)
Age, years: mean (*SD*)	49 (10.2)	47 (9.7)	49 (10.9)
Education			
low (no/primary/low secondary)	6 (18.1)	9 (25.7)	10 (30.3)
medium (high school/low vocational)	12 (36.4)	14 (40.0)	12 (36.3)
high (higher vocational/university)	15 (45.5)	12 (34.3)	11 (33.3)
Depressive symptoms (HDRS): mean (*SD*)	13.5 (5.6)	15.1 (6.9)	15.5 (5.4)
range	2–30	2–30	5–27
Treated in primary care	7 (21)	6 (17)	8 (24)
Bipolar disorder	2 (6)	2 (6)	5 (15)
DSM-IV axis I comorbidity	12 (36)	16 (46)	12 (36)
Psychotherapy yes/no	4 (15)	4 (13)	2 (7)
Use of antidepressant medication^a^			
New	2 (6)	3 (9)	1 (3)
Switch	3 (9)	3 (9)	7 (21)
Maintenance	28 (85)	29 (83)	25 (76)
QALYs, last 12 weeks, mean (*SD*)			
EQ-5D, UK tariff (Dolan) (range − 0.04; 0.23)	0.15 (0.07)	0.14 (0.06)	0.12 (0.07)
Health care costs (last 12 weeks), mean (*SD*)			
Health care use	€2230 (€7186)	€3150 (€4369)	€1999 (€4348)
Medication	€42 (€145)	€18 (€59)	€32 (€79)
Total	€2273 (€7179)	€3168 (€4363)	€2032 (€4342)
Societal (last 12 weeks), mean (*SD*)			
Absence from work	€2541 (€5983)	€2154 (€4852)	€2785 (€5066)
Productivity loss at work	€2156 (€3910)	€2135 (€3486)	€2637 (€4224)
Total (incl. health care costs)	€6828 (€12817)	€7458 (€10201)	€7291 (€9360)

*HDRS* 17-item Hamilton Depression Rating Scale, *QALY* quality adjusted life year, *EQ-5D* EuroQol-5D-3L

^a^New and switch are defined as shorter than 8 weeks on this medication; the rest is maintenance

### Costs at the 32-weeks assessment

Intention-to-treat analysis showed that total costs (over the total 32 weeks; societal perspective) were higher in the ESM-I group (€17,957) than in the control group (€16,216) and the pseudo-intervention group (€16,816; Table 2). However, these differences were not statistically significant after adjustment for baseline costs (Table 2, last two columns; Additional file 3: Table S1). There were also no statistically significant differences between ESM-I and the control groups in any of the cost categories separately (Table 2).

**Table 2:** Costs over 32 weeks (intention-to-treat)

	ESM-I (*n* = 33)	Pseudo-intervention (*n* = 35)	Control (*n* = 33)	Regression coefficients; B (*p*-value obtained from permutation analyses)	
	M (SD)	M (SD)	M (SD)	ESM-I vs Control	ESM-I vs Pseudo-intervention
Health care use (total)^a^	€6751 (€19420)	€6510 (€7315)	€6520 (€14082)	-€165 (*p* = 0.96)	€1818 (*p* = 0.66)
Medication	€104 (€296)	€25 (€56)	€75 (€171)	€12 (*p* = 0.82)	€36 (*p* = 0.54)
Total health costs^b^	€7648 (€19402)	€7225 (€7304)	€6596 (€14072)	€640 (*p* = 0.80)	€1957 (*p* = 0.61)
Societal					
Absence from work	€6067 (€13691)	€4732 (€9372)	€5379 (€11889)	€945 (*p* = 0.74)	€857 (*p* = 0.75)
Productivity loss at work	€4234 (€7721)	€4858 (€8438)	€4241 (€7627)	€605 (*p* = 0.76)	-€594 (*p* = 0.80)
Total	€17957 (€31329)	€16816 (€17595)	€16216 (€27756)	€2483 (*p* = 0.74)	€2152 (*p* = 0.75)

^a^Several types of health care consumption see Additional file 3: Table S1

^b^Including intervention costs in ESM-I and pseudo-intervention group

### HDRS and QALYs at the 32-weeks assessment

At 32 weeks, mean HDRS score was three units lower in the ESM-I group than in the control group (after adjustment for baseline HDRS scores), which was statistically imprecise by conventional alpha (Table 3; B = −3.1, *p* = 0.051, intention-to-treat analysis). The ESM-I and the pseudo-intervention group did not differ (B = −1.13, *p* = 0.47). There was no evidence that ESM-I participants were more often in symptomatic remission compared with control group participants (OR = 2.65, *p* = 0.12); ESM-I participants did not differ in the rate of symptomatic remission compared with the pseudo-intervention participants (OR = 1.84, *p* = 0.29).

**Table 3:** Outcomes (intention-to-treat)

	ESM-I (*n* = 33)	Pseudo-intervention (*n* = 35)	Control (*n* = 33)	Regression coefficients; B (95% confidence interval)^a^	
				ESM-I vs Control	ESM-I vs Pseudo-intervention
HDRS (at 32 weeks)					
HDRS score^a^	10.8 (7.1)	13 (7.12)	15.3 (8.3)	−3.1 (−6.2; 0.01)	−1.13 (−4.18; 1.92)
Improvement since baseline	2.7 (6.1)	2.1 (5.9)	0.24 (7.5)	3.1 (−0.01; 6.2)	1.13 (−1.92; 4.18)
Symptomatic remission^b^	11 (33.3)	9 (25.7)	6 (18.2)	OR = 2.65 (0.79; 8.9)	OR = 1.84 (0.59; 5.7)
QALYs (over 32 weeks)					
EQ-5D, UK tariff	0.45 (0.17)	0.38 (0.18)	0.32 (0.18)	0.08 (0.02; 0.10)^*^	0.04 (−0.02; 0.10)

*HDRS* 17-item Hamilton Depression Rating Scale, *QALY* quality adjusted life year, *EQ-5D* EuroQol-5D-3L

*p < 0.05

^a^Controlled for baseline values

^b^Symptomatic remission = HDRS score ≤ 7

QALYs were higher in the ESM-I group than in the control group (B = 0.08, *p* = 0.01, Table 3), but the difference between ESM-I and pseudo-intervention group was not statistically significant (B = 0.04, *p* = 0.15).

### Cost-effectiveness and cost-utility analysis (time horizon 32 weeks)

**Fig. 2:**
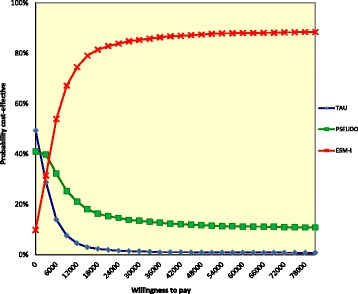
Cost-effectiveness acceptability curve of the base-case analysis, assessing the Hamilton Depression Rating Scale. The analysis was controlled for baseline costs. The lines per treatment indicate the probability (y-axis), i.e., the proportion of replications this treatment has the highest net monetary benefit given various levels of willingness to pay (x-axis). At those willingness-to-pay levels where ESM-I has the higher probability compared with the other two treatments (summing up to 100%), ESM-I is the most cost-effective option. TAU = treatment as usual (control group); PSEUDO = pseudo-intervention group; ESM-I = ESM-intervention group

In the cost-effectiveness analysis (outcome: HDRS), ESM-I had the highest probability of being cost-effective compared with treatment as usual and pseudo-intervention when willingness to pay was over €4000 ranging from a probability of 10 to 86% (when willingness to pay is €0 and €37,500 respectively; Fig. 2). Note that the treatment with the highest probability of cost-effectiveness is the upper line in the figure at each level of willingness to pay.

**Fig. 3:**
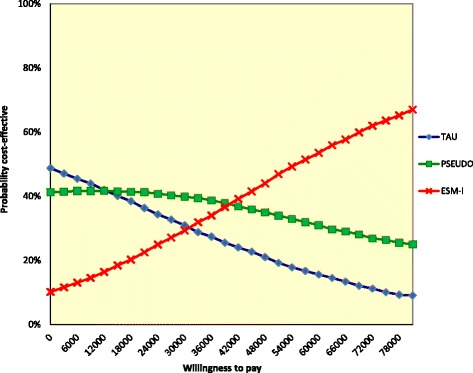
Cost-effectiveness acceptability curve of the base-case analysis, assessing the EQ-5D. The analysis was controlled for baseline costs. The analysis was controlled for baseline costs with the bootstrapped societal costs and EQ-5D-based QALYs. The lines per treatment indicate the probability (y-axis), i.e., the proportion of replications this treatment has the highest net monetary benefit, given various levels of willingness to pay (x-axis). At those willingness-to-pay levels where ESM-I has the higher probability compared with the other two treatments (summing up to 100%), ESM-I is the most cost-effective option. At the willingness-to-pay threshold of €50,000, the probability is 46% for ESM-I, which is higher compared with TAU (20%) and Pseudo (34%). TAU = treatment as usual (control group); PSEUDO = pseudo-intervention group; ESM-I = ESM-intervention group

In the cost-utility analysis (outcome: QALYs), the CEAC curve showed that ESM-I had the highest probability of being the most optimal of the three treatments when willingness to pay was over €40,500 (Fig. 3). At the a priori willingness-to-pay threshold of €50,000, ESM-I was the intervention with the highest probability of being cost effective (ESM-I 46%, pseudo-intervention 34%, treatment as usual 20%).

### Sensitivity analyses

Table 4 presents both the base-case and sensitivity cost-effectiveness and cost-utility results. When willingness to pay levels were higher than between €30,000 and €40,000, ESM-I was the most optimal treatment (Table 4, Fig. 2, Additional file 4: Figure S1, Additional file 5: Figure S2, Additional file 6: Figure S3 and Additional file 7: Figure S4) in the cost effectiveness analyses (HDRS). The sensitivity analysis from the health care perspective and the complete cases analysis were more optimistic than the base-case analysis, being most cost effective from €3000 and €3750, respectively.

**Table 4:** Results of base-case and sensitivity analyses of the cost-utility and cost-effectiveness analyses: willingness to pay when compared with both control conditions

	Level of willingness to pay when ESM-I is more cost effective than		Percentage at WtP threshold^a^
	treatment as usual	pseudo-intervention	
HDRS	€	€	
Base-case analysis	3000	4000	
Different GP cost calculation	3000	4000	
Health care perspective	1500	3750	
Complete cases	2250	3000	
NOT controlling for baseline costs	1250	0^b^	
QALY	€	€	%
Base-case analysis	31, 500	40, 500	46%
Dutch instead of UK tariff	32, 500	43, 000	44%
Different GP cost calculation	30, 500	39, 500	45%
Health care perspective	16, 500	36, 000	64%
Complete cases	23, 500	30, 500	65%
NOT controlling for baseline costs	11, 500	0^b^	58%

^a^The probability that ESM-I is most cost-effective at the willingness-to-pay threshold of €50,000

^b^A value of €0 indicates that ESM-I is dominant compared with the other intervention, i.e., ESM-I shows more improvement in outcomes at lower costs compared with the other treatment

**Fig. 4:**
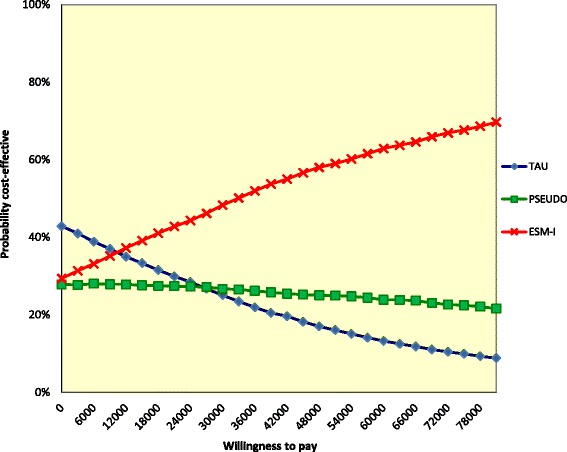
Cost-effectiveness acceptability curve of a sensitivity analysis assessing the EQ-5D: unadjusted for baseline costs

At the willingness-to-pay threshold of €50,000, the probability that ESM-I is most cost-effective was between 44 and 65% (cost utility analysis, Table 4, Figs. 3 and 4 and Additional file 8: Figure S5, Additional file 9: Figure S6, Additional file 10: Figure S7 and Additional file 11: Figure S8). Again, the sensitivity analysis from the health care perspective and the complete cases analysis were most optimistic with percentages of 64 and 65% at the willingness-to-pay threshold of €50,000.

## Discussion

### Key findings

The present study provides, to our knowledge, the first economic evaluation of an intervention using ESM in patients with major depression. The results suggest that ESM-I is more expensive, but also more clinically effective than both treatment as usual and pseudo-intervention.

In the cost-effectiveness analysis and cost-utility analysis, ESM-I was the most optimal strategy when willingness to pay was over €3000 and €40,500, respectively. All sensitivity analyses except one were similar to the base-case analysis. That one exception, that is the analysis unadjusted for baseline costs, had lower willingness to pay, and a probability of cost-effectiveness at €50,000 of 58%. In addition, CEAC showed that ESM-I cost-effectiveness probability increased rapidly towards the most favourable treatment.

Furthermore, although costs are below the threshold set for a QALY (€50,000), such a threshold could not be defined for the HDRS. Therefore, we can only tentatively conclude that ESM-I is cost-effective.

### Cost-effectiveness of ESM-I in real life major depression treatment

The present trial shows that ESM-I consisting of protocolled feedback delivered by a researcher has the potential to be cost-effective. When implementing ESM-I in real life treatment, feedback can be delivered directly to the patient and professional caregiver. Feasibility and cost-effectiveness are hypothesized to increase when the option of feedback provided by a third person (the researcher) is replaced with ESM-I feedback that forms an integral part of the treatment. ESM-I could then also be used to enrich psychological treatments such as cognitive behaviour therapy [52] with daily life contextual information and to bring that therapy out of the mental health care setting into daily life. Our six-week ESM intervention has been shown feasible in outpatients with major depression [17], but the feasibility of implementation in routine clinical practise is not yet established [13].

Web-based feedback systems for ESM-I applications are under development. If such a web-based system allows individuals to navigate through their own feedback, this may facilitate implementation of the current ESM intervention by promoting easy access to and flexible use of feedback for patients as well as professional caregivers. This should be backed up by appropriate resources for professional caregivers including training, monitoring, and technical support [53]. In addition, withdrawal of the professional caregiver and patient disengagement may be an important issue, requiring research to improve sustained use [54].

### Effects of ESM-I on depressive symptoms

The ESM-I group showed lower HDRS scores at 32-weeks than the two control groups, suggesting that ESM-I reduced depressive symptoms. However, although the economic evaluation showed that ESM-I may be cost-effective, in the accompanying regression analyses (HDRS and QALYs; Table 3), the difference between the ESM-I and the pseudo-intervention group was not statistically significant while the difference between ESM-I and control group was statistically imprecise by conventional alpha. The effect study, accompanying the present economic evaluation [17], did show that allocation to ESM-I was associated with a statistically significant linear decrease in HDRS depressive symptoms over time that lasted throughout the study. This decrease was significantly stronger than in the control group to a degree that can be considered clinically relevant (difference > 3 HDRS units; [46, 47]). The difference with the pseudo-experimental group was clinically relevant and borderline significant [17]. For the regression analysis results accompanying the cost effectiveness results in the present paper, less data were used than in the original analyses which included all follow-up assessments. In addition, the original paper analysed subjects as randomized with available data while the present paper imputed data (using last observation carried forward).

### Cost-effectiveness and severity of depression

The study sample consisted of patients with a major depression with current symptoms in the mild to severe range, including residual depressive states. Given that meta-analytic evidence suggests that the efficacy of psychotherapeutic interventions may be larger in patients with higher levels of pre-treatment depressive symptoms [55], a subgroup analysis only including patients with severe or very severe depressive symptoms was warranted. However, in the present data, the number of patients in subgroups (e.g. only 20 patients with HDRS ≥ 19) was too low to obtain valid results. Future economic evaluations of ESM-I should include sufficient numbers of patients at each level of severity to enable subgroup analysis in patients with mild/moderate and with severe/very severe symptoms separately.

### Methodological considerations

The present study was limited to patients aged between 18 and 65 years (mean age 48 years) and more than 90% of the sample was from Dutch origin. ESM-I is designed to obtain insights in everyday life and, therefore, we recruited outpatients that could engage in ESM self-monitoring in their home environment. Outpatients were included in the study if they scored above remission level (HDRS > 7) at study entrance. This mild inclusion criterion, coupled with the time intensive nature of the study protocol (multiple visits to the researcher on top of an intensive intervention consisting of 6 weeks of self-monitoring), may have led to recruiting mainly participants in a mild to moderate depressive state. However, this may be a rather accurate representation of the population of patients with major depression, of which the majority experiences mild to moderate symptoms, and using higher HDRS cut-offs would compromise the external validity of the trial [56, 57]. On the other hand, our sample was mostly recruited from specialised mental health care settings (approximately 20% was treated in primary care only), and had a diagnosis of major depression as well as current symptoms for which they were using antidepressants. Although the results may not be generalizable to all outpatients with major depression, they may be generalizable to outpatients with complex mental problems who are using antidepressants.

The present paper has several limitations. First, owing to the nature of the intervention, it was not possible to blind participants and the use of envelopes could potentially have led to biased allocation. However, given that care-providers were not involved in the randomization process and most envelopes were drawn from a distance, with one researcher drawing an envelope for another researcher, it is unlikely that subversions to the procedure took place. Researchers conducting the post-intervention assessments were also not blind to treatment allocation due to resource constraints. Thus findings may reflect a placebo response. However, the effect study [17] showed that directly after the six-week intervention, the decrease in HDRS ratings was similar in the ESM-I group and the pseudo-intervention group, while in the pseudo-intervention group effects did not appear to persist during the full 32-weeks of the trial. It is often assumed that placebo effects in depression do not persist in the long run [58]. Although, this belief has been falsified [58], the difference in persistence between the pseudo-intervention group and the ESM-I group may evidence that it is unlikely that our findings are completely attributable to a placebo effect. The improvement in the ESM-I group showed a persistent, steady and clinically relevant growth over time in the full 32 weeks, further making the possibility of a placebo effect even more unlikely.

Second, all three treatment arms were embedded in an extensive research protocol, including regular assessment of depressive symptoms and two five-day ESM assessments. Besides treatment effects, patients may have had non-specific benefits from self-monitoring. Therefore, what has been called treatment as usual in the present paper, strictly is not. ESM-I may be even more cost-effective when compared with true treatment as usual.

Third, we used the human capital method rather than the friction costs method to calculate work absence costs, because the PRODISQ absenteeism module only asked number of absent days during a period of 3 months, while friction period was longer at the time of data collection (approximately 5 months) [37]. Therefore, end of the friction period could not be identified.

Fourth, sampling uncertainty was estimated using the non-parametric bootstrapping approach. Alternatively, another common approach for the handling of trial-based data would have been to estimate the mean total costs per treatment condition using a GLM that assumes a Gamma distribution for costs (i.e., to accommodate the skewness in the distribution of costs). This would also allow for the regression-based adjustment of cost estimates through the inclusion of possible covariates in the GLM. It could therefore be considered a limitation that non-adjusted costs were reported.

Fifth, sample sizes for the present study were rather small. Results need to be replicated in studies with larger sample sizes. However, other economic evaluations are also performed using small sample sizes. Sensitivity analyses and bootstrapping are required to correct for sampling uncertainty and to prevent chance findings. As expected, costs were not normally distributed and, therefore, a condition for regression was not met (normal distribution of the residuals). We therefore performed non-parametric bootstrap resampling. However, baseline costs were also skewed and had outliers, and regressing baseline costs onto total costs [49] resulted in non-normal distribution of residuals, even after transformation to the natural logarithm. Several methods to deal with the problem of outliers have been advocated [49]. However, removing various percentages (2, 5, 10, 20, or 30%) of observations at the extremes, resulted in non-normally distributed residuals and in inconsistent regression coefficients of baseline costs (B = 0.84, 0.82, 0.79, 0.72, 0.66, respectively; base-case analyses: B = 0.86). Therefore, the best option to correct for baseline costs [49] was impossible in the present data, and it is most prudent to perform the delta method to control for baseline costs rather than regression-based adjustment [49] (see also Methods).

Furthermore, we chose for easy methods to deal with missing data because the number of missings was limited. The proportion of missing values was not significantly associated with treatment allocation, nor with baseline and previous observed depression scores or baseline demographics. Last observation carried forward, next observation carried backward, and mean imputation have been shown to perform as good as multiple imputation [59].

Finally, of all parameters that we varied in the sensitivity analyses, correction for baseline costs was the only factor that changed the willingness to pay, but probability of cost-effectiveness at the a priori threshold of €50,000 remained similar to the base-case analysis. Correction for baseline costs is relatively new in economic evaluations, in contrast to epidemiology and statistics, were controlling for baseline differences is standard practise to get valid results [60]. The present results show that the impact of controlling for baseline may be considerable and suggest that, as in other fields of research, results without baseline correction may be invalid.

## Conclusion

We may tentatively conclude that ESM-I is cost-effective in outpatients with major depression. Only tentatively because the probability that ESM-I was cost effective was only 44% at the predefined threshold of €50,000, while no threshold for the HDRS could be defined.

Future studies are needed to replicate the present findings and to study patients with severe depressive symptoms separately. If future research replicates effectiveness and cost-effectiveness, we would recommend ESM-I as an addition to psychopharmacological treatment as usual. Integration of ESM-I in psychological treatment is also a possibility.

## Additional files

Additional file 1: CONSORT Checklist. (DOC 217 kb)

Additional file 2: Protocol. Original study protocol. (PDF 245 kb)

Additional file 3: **Table S1.** Costs at baseline and costs over 32 weeks (intention-to-treat) per type of consultation. **Table S2.** Unit costs per cost category. Costs were obtained from a Dutch cost manual (2009-prices, Hakkaart van Roijen 2010) and calculated to their 2012 value (Statline). (DOCX 20 kb)

Additional file 4: Figure S1. Cost-effectiveness acceptability curve assessing HDRS, sensitivity analysis: GP costs based on psychiatric tariff. (DOCX 93 kb)

Additional file 5: Figure S2. Cost-effectiveness acceptability curve assessing HDRS, sensitivity analysis: health care perspective. (DOCX 94 kb)

Additional file 6: Figure S3. Cost-effectiveness acceptability curve assessing HDRS, sensitivity analysis: completers only. (DOCX 93 kb)

Additional file 7: Figure S4. Cost-effectiveness acceptability curve assessing HDRS, sensitivity analysis: unadjusted for baseline costs. (DOCX 95 kb)

Additional file 8: Figure S5. Cost-effectiveness acceptability curve, sensitivity analysis, assessing EQ-5D: Dutch valuation of the EQ-5D. (DOCX 96 kb)

Additional file 9: Figure S6. Cost-effectiveness acceptability curve, sensitivity analysis, assessing EQ-5D: GP costs based psychiatric tariff. (DOCX 92 kb)

Additional file 10: Figure S7. Cost-effectiveness acceptability curve, sensitivity analysis, assessing EQ-5D: health care perspective. (DOCX 96 kb)

Additional file 11: Figure S8. Cost-effectiveness acceptability curve, sensitivity analysis, assessing EQ-5D: completers only. (DOCX 95 kb)

## Abbreviations

CEAC: Cost-effectiveness acceptability curve
DSM-IV: Diagnostic and statistical manual of mental disorders fourth edition
EQ-5D: EuroQol-5D-3L
ESM: Experience sampling method
ESM-I: Experience sampling method intervention
HDRS: Hamilton depression rating scale
PRODISQ: Productivity and disease questionnaire
QALY: Quality adjusted life years
TAU: Treatment as usual
TiC-P: Trimbos/Institute for Medical Technology Assessment questionnaire for costs associated with psychiatric illness
