# Supplementary material for: Economic evaluation of an experience sampling method intervention in depression compared with treatment as usual using data from a randomized controlled trial
Source: BMC Psychiatry. 2017 Dec 29;17:415. doi: 10.1186/s12888-017-1577-7 (PMC5747107; doi:10.1186/s12888-017-1577-7)
Supplement: Supplementary file 3 — Table S1. Costs at baseline and costs over 32 weeks (intention-to-treat) per type of consultation. Table S2. Unit costs per cost category. Costs were obtained from a Dutch cost manual (2009-prices, Hakkaart van Roijen 2010) and calculated to their 2012 value (Statline). (DOCX 20 kb) [file 12888_2017_1577_MOESM3_ESM.docx]

**S1 Table.** Costs at baseline and costs over 32 weeks (intention-to-treat) per type of consultation.

| **Baseline** | **ESM-I**  **(n=33)** | **Pseudo-intervention**  **(n=35)** | **Control group**  **(n=33)** |
| --- | --- | --- | --- |
| General practitioner  Community centre for ambulatory mental health care  Psychiatrist, psychologist, or psychotherapist in private practice  Psychiatrist, psychologist, or psychotherapist in hospital as outpatient  Industrial physician | 58 (58)  776 (2829)  229 (385)  215 (512)  30 (53) | 76 (99)  1239 (2171)  272 (668)  235 (506)  13 (23) | 87 (134)  402 (643)  278 (336)  270 (581)  17 (28) |
| Medical specialist  Paramedical health care worker  Social worker  Clinic for alcohol and drugs  Alternative healer | 42 (79)  38 (96)  10 (43)  0 (0)  47 (135) | 76 (172)  45 (143)  49 (160)  0 (0)  11 (50) | 125 (188)  41 (98)  21 (80)  0.9 (5.1)  10 (35) |
| Self-help group  Day or part-time treatment  Admitted to health care institution | 22 (99)  317 (1708)  448 (2573) | 16 (77)  655 (1867)  462 (2363) | 1.7 (9.7)  59 (289)  686 (3858) |

| **24 Weeks (excluding the 8 week intervention period)** | **Experimental**  **(n=33)** | **Pseudo-experimental**  **(n=35)** | **Control**  **(n=33)** |
| --- | --- | --- | --- |
| General practitioner  Community centre for ambulatory mental health care  Psychiatrist, psychologist, or psychotherapist in private practice  Psychiatrist, psychologist, or psychotherapist in hospital as outpatient  Industrial physician | 82 (68)  1829 (5864)  401 (684)  149 (304)  31 (70) | 90 (85)  1577 (1920)  272 (503)  383 (897)  20 (47) | 130 (178)  759 (1280)  484 (747)  294 (639)  38 (88) |
| Medical specialist  Paramedical health care worker  Social worker  Clinic for alcohol and drugs  Alternative healer | 139 (291)  79 (169)  31 (90)  0 (0)  71 (243) | 182 (390)  75 (271)  114 (434)  0 (0)  25 (70) | 234 (334)  226 (436)  153 (865)  177 (10)  18 (70 |
| Self help group  Day or part-time treatment  Admitted to health care institution | 5 (21)  1198 (3769)  1148 (5200) | 26 (116)  1536 (3910)  312 (1647) | 12 (50)  65 (371)  2774 (11595) |

| **8 week intervention period^1^** | **Experimental**  **(n=33)** | **Pseudo-experimental**  **(n=35)** | **Control**  **(n=33)** |
| --- | --- | --- | --- |
| General practitioner  Community centre for ambulatory mental health care  Psychiatrist, psychologist, or psychotherapist in private practice  Psychiatrist, psychologist, or psychotherapist in hospital as outpatient  Industrial physician | 35 (28)  577 (1908)  161 (248)  101 (223)  16 (29) | 41 (40)  669 (926)  144 (264)  131 (229)  8 (14) | 47 (63)  251 (368)  176 (196)  123 (246)  11 (19) |
| Medical specialist  Paramedical health care worker  Social worker  Clinic for alcohol and drugs  Alternative healer | 43 (104)  24 (54)  10 (29)  0 (0)  30 (88) | 52 (107)  32 (111)  33 (95)  0 (0)  9 (27) | 79 (102)  55 (99)  33 (146)  0.6 (3.4)  5 (15) |
| Self help group  Day or part-time treatment  Admitted to health care institution | 7 (33)  284 (1163)  299 (1715) | 7 (27)  519 (1358)  252 (942) | 4 (17)  20 (96)  527 (1847) |

^1^ Presented separately, because estimated using mean imputation

**Table S2.** Unit costs per cost category.

|  | 2009 € | 2012 €^1^ |
| --- | --- | --- |
| **Outpatient** |  |  |
| General practitioner | 28 | 29.73 |
| General practitioner for mental health contact | 57 | 60.53 |
| Outpatient mental health | 171 | 181.58 |
| Psychologist/psychiatrist (community centre for ambulatory mental health care) | 103 | 109.37 |
| **Outpatients’ clinic** |  |  |
| General or academic hospital, outpatient | 171 | 181.58 |
| Psychotherapy institute | 173 | 183.70 |
| Psychotherapy | 173 | 183.70 |
| Average policlinic | 172 | 182.29 |
| **Other** |  |  |
| Industrial physician | 28 | 29.73 |
| Medical specialist | 72 | 76.45 |
| Paramedical healthcare worker | 29.40 | 31.22 |
| Social worker | 65.00 | 69.02 |
| **Day care** |  |  |
| General or academic hospital, day care | 251 | 266.53 |
| Psychotherapy, day care | 171 | 163.53 |
| Psychiatric hospital, day care | 171 | 163.53 |
| Other hospital | 202.5 | 215.03 |
| **Admission** |  |  |
| Academic hospital, admission day | 575 | 610.58 |
| General hospital, admission day | 435 | 461.91 |
| Psychotherapy admission | 232 | 246.35 |
| Psychiatric hospital admission | 232 | 246.35 |
| Other hospital | 414 | 439.61 |

Costs were obtained from a Dutch cost manual (2009-prices, Hakkaart van Roijen 2010) and calculated to their 2012 value (Statline).

^1^ 2012 prices are obtained from 2009 prices by multiplying by 106.1878%
