# Supplementary material for: Economic evaluation of an experience sampling method intervention in depression compared with treatment as usual using data from a randomized controlled trial
Source: BMC Psychiatry. 2017 Dec 29;17:415. doi: 10.1186/s12888-017-1577-7 (PMC5747107; doi:10.1186/s12888-017-1577-7)
Supplement: Supplementary file 3 [file 12888_2017_1577_MOESM10_ESM.docx]

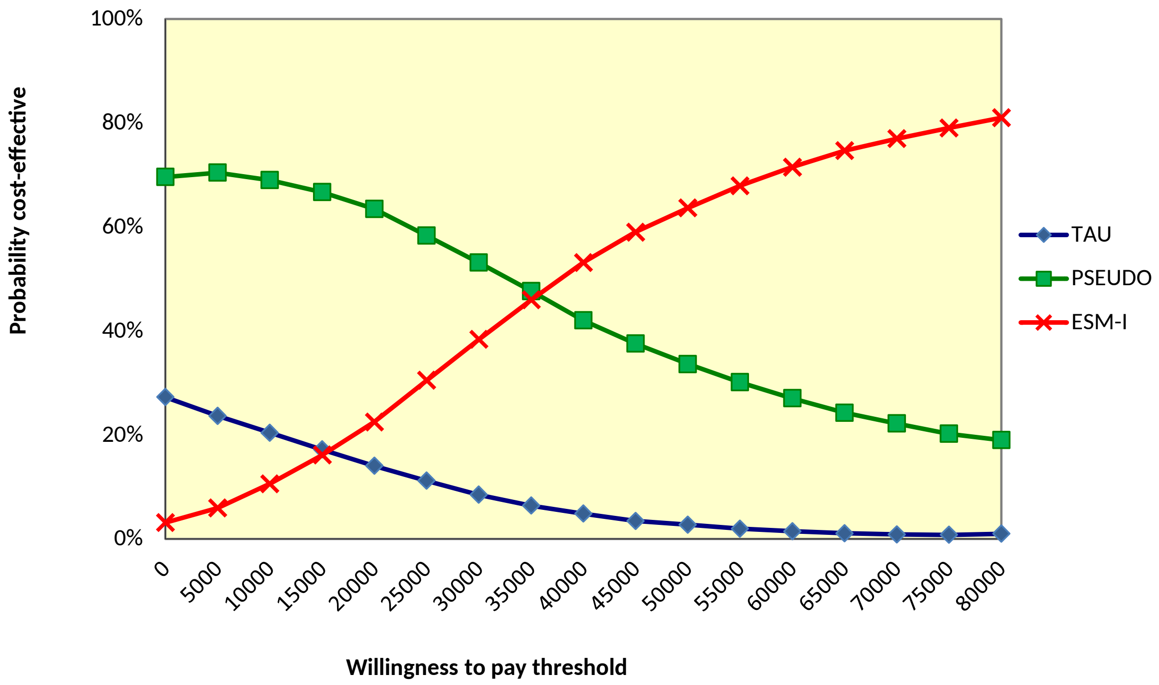

Probability cost-effective
100%
80%
60%
40%
20%
0%
0
5000
10000
15000
20000
25000
30000
35000
40000
45000
50000
55000
60000
65000
70000
75000
80000
Willingness to pay threshold
TAU
PSEUDO
ESM-I
